# A Focused Review of the Initial Management of Patients with Acute Respiratory Distress Syndrome

**DOI:** 10.3390/jcm12144650

**Published:** 2023-07-13

**Authors:** Arunee Motes, Tushi Singh, Noella Vinan Vega, Kenneth Nugent

**Affiliations:** Department of Internal Medicine, Texas Tech University Health Sciences Center, Lubbock, TX 79430, USA; arunee.motes@ttuhsc.edu (A.M.); tushi.singh@ttuhsc.edu (T.S.);

**Keywords:** acute respiratory distress syndrome, pathogenesis, treatment, phenotypes, precision medicine

## Abstract

At present, the management of patients with acute respiratory distress syndrome (ARDS) largely focuses on ventilator settings to limit intrathoracic pressures by using low tidal volumes and on FiO_2_/PEEP relationships to maintain optimal gas exchange. Acute respiratory distress syndrome is a complex medical disorder that can develop in several primary acute disorders, has a rapid time course, and has several classifications that can reflect either the degree of hypoxemia, the extent of radiographic involvement, or the underlying pathogenesis. The identification of subtypes of patients with ARDS would potentially make precision medicine possible in these patients. This is a very difficult challenge given the heterogeneity in the clinical presentation, pathogenesis, and treatment responses in these patients. The analysis of large databases of patients with acute respiratory failure using statistical methods such as cluster analysis could identify phenotypes that have different outcomes or treatment strategies. However, clinical information available on presentation is unlikely to separate patients into groups that allow for secure treatment decisions or outcome predictions. In some patients, non-invasive positive pressure ventilation provides adequate support through episodes of acute respiratory failure, and the development of specialized units to manage patients with this support might lead to the better use of hospital resources. Patients with ARDS have capillary leak, which results in interstitial and alveolar edema. Early attention to fluid balance in these patients might improve gas exchange and alter the pathophysiology underlying the development of severe ARDS. Finally, more attention to the interaction of patients with ventilators through complex monitoring systems has the potential to identify ventilator dyssynchrony, leading to ventilator adjustments and potentially better outcomes. Recent studies with COVID-19 patients provide tentative answers to some of these questions. In addition, expert clinical investigators have analyzed the promise and difficulties associated with the development of precision medicine in patients with ARDS.

## 1. Introduction

Mechanical ventilation provides life-saving support for patients who have acute respiratory failure. Clinicians will need to decide about the cause of the respiratory failure, the degree of respiratory failure based on arterial blood gases, the best ventilator support mode, and the most effective drug therapy to treat the cause of the respiratory failure and to maintain safe ventilation. Day-to-day care requires the management of electrolytes, fluid balance, nutrition, and prophylactic measures to prevent deep venous thrombosis, gastric ulceration, and muscle wasting. The management and outcome will depend on the cause of the acute respiratory failure and the expected response to medication. For example, patients with acute respiratory failure secondary to severe asthma will likely have better outcomes than patients with severe acute respiratory distress syndrome (ARDS). In all cases, the clinician will need to monitor patient–ventilator interaction, fluid balance, and patient comfort during ventilation.

The admission of patients with acute hypoxemic respiratory failure who have ARDS or are at risk for developing ARDS creates several important immediate problems. Clinicians will likely revisit the diagnostic information and management decisions repeatedly over the first 48 h in the ICU. Does better characterization of the patient’s respiratory disorder based on phenotypes or endotypes result in better treatment decisions and outcomes? Can precision medicine provide a more exact diagnosis or classification and lead to personalized care? If the diagnostic methods needed to establish an endophenotype classification are not available, then the clinician must depend on clinical trial results to initiate treatment. The initial management decisions might ask the following questions. Can non-invasive ventilation provide adequate support for patients with acute respiratory failure? Can early attention to fluid balance improve outcomes in patients with acute respiratory failure even if they do not have cardiogenic pulmonary edema? Does more complete monitoring of the patient–ventilator interaction provide useful information about ventilator adjustments? The answers to these questions will require a detailed analysis of the patient and will be considered in this review.

## 2. An Overview of ARDS and Precision Medicine

### 2.1. Background

Acute respiratory distress syndrome is a complex and heterogeneous disorder, and multiple primary events can result in acute lung injury and ARDS [1,2]. These patients can have different levels of hypoxemia, different distributions of opacities on chest radiographs, and different types of extra-pulmonary organ dysfunction. The pathologic changes in the lung depend on the primary injury, the development of inflammatory responses, and the timeframe and trajectory of acute events. The goal of precision medicine and personalized care involves the identification of the underlying endotype and the subsequent phenotype, which in turn potentially provides a more focused treatment plan. Ideally, this occurs during the first 48 h after admission. However, this approach is difficult in patients with ARDS, given the rapid evolution of lung injury and the limited time to collect specialized diagnostic information and to make decisions regarding the best treatment.

Patients with acute respiratory distress syndrome have a mortality rate of 30–46% [3]. The Berlin definition requires exposure to a known risk factor or worsening of the respiratory symptoms within one week and bilateral infiltrates on imaging that are not completely explained by cardiogenic pulmonary edema [4]. Its severity is classified as mild, moderate, or severe based on the degree of hypoxemia as measured by PaO_2_/FiO_2,_ while on a minimum of 5 cm H_2_O of positive end-expiratory pressure (PEEP). The injury in ARDS involves the alveolar epithelium and or the capillary endothelium [1]. This injury results in increased permeability of alveolar capillary barriers, decreases alveolar fluid clearance, disrupts surfactant function in alveoli, and activates both inflammation and coagulation in the lung. Epithelial injury biomarkers, such as the receptor for the advanced glycation end products (RAGE) and surfactant protein D (SP-D), are increased in patients with direct lung injuries, and endothelial injury biomarkers, such as angiopoietin 2 (Ang-2), are increased in patients with indirect lung injuries, e.g., associated with pancreatitis [5,6].

The initial stage in ARDS is an exudative phase, characterized by activation of the innate immune cells, which damage endothelial and epithelial cells and by the accumulation of interstitial and alveolar fluid. The next stage involves repair processes to restore cellular barrier function; the final fibrotic stage occurs in some ARDS patients who develop irreversible fibrosis. The factors that drive this process through distinct ARDS stages are unknown, and treatment responses in ARDS likely depend on the time frame for the injury and ongoing acute responses. The pathologic changes in ARDS include diffuse alveolar damage (DAD) with destruction of alveolar structures, the development of hyaline membranes, the infiltration of leukocytes, and the deposition of fibrin; these histologic changes are present in post-mortem studies in approximately 45% of patients who meet the current clinical definition of ARDS [7,8]. Pneumonia without hyaline membranes or DAD is the second most common histologic finding in autopsy studies. However, classification based on the primary source and location of the injury may oversimplify the complexity of this syndrome, since ICU patients are frequently exposed to multiple possible insults, including volutrauma and barotrauma [9,10,11].

The complex pathophysiology and the lack of established drug therapy for patients with ARDS has led investigators to try to analyze this syndrome using precision medicine.

### 2.2. Precision Medicine

The essential elements necessary for precision medicine include phenotyping, endophenotyping, and genomic profiling [12]. The sequence of events starts with a genomic network, which results in transcription and the development of proteins. These proteins provide the basis for metabolism. Eventually, whole organ effects, including a psychosocial network, and clinical phenotypes develop. This process can be modified at the post-genomic level by modification of translation and metabolic events. In addition, there can be epigenomic modifications and environmental exposures and microbiome interactions likely contribute to this process. The trajectory for these events will depend on whether the disorder is an acute medical disorder or a chronic medical disorder. Intervention will require a comprehensive understanding of the fundamental cause(s) of a clinical disorder, which could be at the genomic level, the transcription level, the proteomic level, or the metabolomic level. Proteomics is a complete evaluation of the function and structure proteins, including interactions, function, structure, and cellular activities. This can provide information about their basic function and alterations in protein function associated with disease. Metabolomics is the large-scale study of small molecules commonly known as metabolites within cells, body fluids, tissues, and organisms. This provides insight into the metabolic activity of cells during injury and repair.

Battaglini et al. have reviewed personalized medicine using all omics approaches in acute respiratory distress to identify biological subtypes [13]. Their review considered genomics, transcriptomics, proteomics, and metabolomics. These studies have complex technological requirements. At a more simple but fundamentally important level, investigators need to decide which tissue or fluid to study. Studies in patients with acute lung disease could use bronchoalveolar fluid, plasma, lung biopsies, and exhaled condensates. These studies could identify genes that predispose patients to acute severe lung injury, changes in protein synthesis or catabolism associated with severe injury, or metabolic pathways associated with injury and repair. This information could potentially lead to the development of drugs, which inhibit the relevant biological process or enhance repair.

Precision medicine has the potential to improve outcomes by matching treatment to individuals or subgroups of patients most likely to benefit from a particular treatment [1]. The introduction of precision medicine into the management of ARDS requires a comprehensive understanding of the complex heterogeneity of this syndrome and identifying methods to predict treatment responses in individual patients. These clinical trials will likely involve individualized strategies based on levels of biomarkers, suggesting a particular dominant (or important) pathophysiologic mechanism of injury. However, multiple difficulties limit the use of recently identified biomarkers of epithelial and endothelial injury (RAGE and Ang-2), vascular leak (BAL albumin), and the innate inflammatory response (IL-6, IL-8, and tumor necrosis factor alpha) in animal studies and in pilot studies in patients with ARDS [6,14]. Most of these biomarkers were identified during pre-clinical studies and are not usually measured during routine clinical practice. These pre-clinical results frequently depend on the analysis of bronchoalveolar lavage (BAL) samples; however, ARDS research in ICUs often depends on plasma samples, which do not fully reflect the inflammatory response in the lungs. In addition, the variable fluid recovery during BAL limits the interpretation of biomarker concentrations. An additional barrier to the use of precision medicine in ARDS includes the rapid time course of this disease process. This syndrome evolves over hours to days, patients are often too sick to undergo biopsies or other invasive diagnostic testing, and test results are necessarily delayed to allow laboratory processing. For diagnostic tests to help identify ARDS phenotypes, clinicians must have the results available quickly.

**Figure 1:**
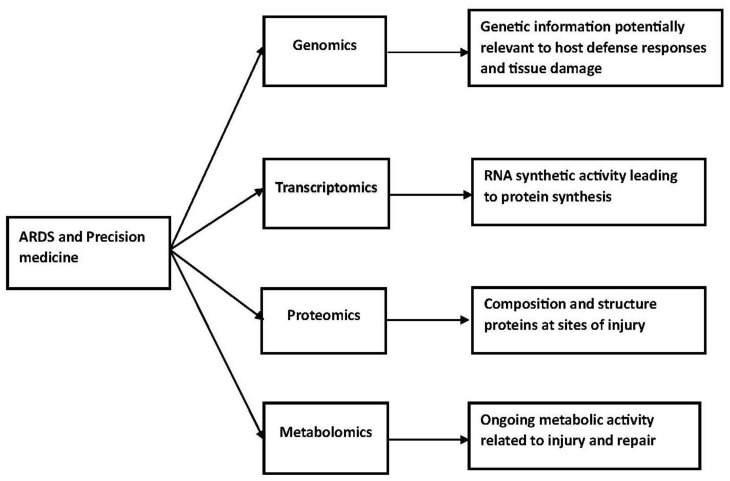
ARDS and precision medicine.

The difficulties associated with developing a precision medicine approach in the management of acute respiratory distress syndrome are numerous and substantial. Most studies to date have focused on the clinical characterization of these patients and the development of clinical trials based on the pathophysiologic characteristics of this disorder. In patients with ARDS, this information will need to be available very early in the hospital course, and effective intervention will need to take place within the first several days of patient management (Figure 1).

### 2.3. Possible Phenotypes in ARDS

Based on the results of the ARDSNet and Low tidal Volume and elevated End-expiratory volume to Obviate Lung Injury trials, two ARDS phenotypes were identified, a phenotype called “hyper-inflammatory”, defined by higher plasma concentrations of inflammatory cytokines, lower serum bicarbonate levels, and higher vasopressor requirements, and a phenotype called “hypo-inflammatory”, defined by lower concentrations of inflammatory cytokines, higher serum bicarbonate levels, and lower vasopressor requirements [15]. Post-hoc analysis of additional ARDS randomized controlled trials has demonstrated that these phenotypes have different responses to randomized treatments, results that were obscured in the original clinical trials that analyzed all ARDS patients as a single group. Patients with the hyper-inflammatory phenotype had lower mortality with a higher PEEP strategy, liberal fluid management, and simvastatin, whereas patients with the hypo-inflammatory phenotype either did not respond or had higher mortality with same treatments [16].

In addition to the biochemical heterogeneity in the injuries in ARDS, there are also significant anatomic and physiologic differences that contribute to the complexity of this disorder. Different patient-specific characteristics, such as increased body mass indices, and different injury specific characteristics, such as the distribution of pulmonary opacities, can substantially change lung mechanics and may affect the optimal mechanical ventilation strategy. Consequently, additional research has led to the classification of radiographic subtypes of ARDS, defined as non-focal/diffuse and focal/lobar. Non-focal/diffuse ARDS occurs more frequently after systemic insults that indirectly cause lung injury, is associated with lower levels of RAGE (which reflects epithelial damage), and causes worse lung compliance and higher mortality. The identification of the distinct radiographic phenotypes led to trials, which attempted to personalize mechanical ventilation strategies with the hypothesis that individuals with focal/lobar ARDS have an increased volume of normal lung parenchyma, and thus would tolerate higher tidal volumes than those with non-focal/diffuse ARDS, but this trail did not find any change in mortality using radiographic criteria to adjust PEEP [17].

Bos and Ware reviewed the causes, pathophysiology and phenotypes in patients with ARDS in 2022 [18,19]. They discussed subphenotypes based on cause, biological subphenotypes, radiological subphenotypes, and physiological subphenotypes and noted that mechanical ventilation can cause lung injury and inflammation. The development of barotrauma, volutrauma, and biotrauma associated with mechanical ventilation could change the individual patient’s phenotype and make it more difficult to study outcomes. In a second review, Bos et al. considered the various types of alveolar injury in ARDS and the biomarkers available to identify these injuries [10,18]. They suggested that the predominant patterns include epithelial and/or endothelial injury, protein-rich pulmonary edema, and systemic or alveolar inflammatory responses. The most readily available biomarkers would involve bronchoalveolar lavage studies, and analysis of this fluid could identify increased total protein concentrations, increased neutrophil numbers, and increased cytokine levels. Whether or not this information would lead to classifications that predict therapeutic responses is uncertain.

Alipanah et al. also reviewed the possible classification of patients with ARDS using phenotypes [20]. Potential phenotypes would include clinical phenotypes based on potential causes and radiographic presentations, physiologic phenotypes based on PaO_2_/FiO_2_ ratios, and biological phenotypes based on biomarkers reflecting injury and/or inflammation. Classification using these parameters may eventually determine clinically important phenotypes, but this research is limited by the lack of prospective validation. Gorman et al. reviewed ARDS and discussed the diagnosis, outcomes, long-term sequelae, and management [21]. They noted that long-term outcomes in survivors often included physical, mental, and cognitive deficits. Therefore, treatment protocols may need to evaluate both short-term benefits and long-term benefits and consider the possibility that there are survivor phenotypes. Beitler et al. published a position paper on advancing precision medicine for ARDS based on the workshops sponsored by the United States National Heart, Lung, and Blood Institute [22]. They noted that multi-center observational cohort studies were needed to collect information relevant to establishing phenotypes in this syndrome and that rapid diagnostic assays were essential to provide predictive and prognostic enrichment in any trial. Finally, they noted that platform trials with a master protocol with or without adaptive features could accelerate the identification of treatment-responsive subgroups.

The idea of ARDS classification based on phenotypes may have great promise but realistically is not available at present. Heijen et al. analyzed inflammatory biomarkers in mini-BAL fluid aspirations from patients with ARDS and the key features of the lung microbiome in this fluid [23]. Patients were classified into three separate groups with two subphenotypes in each group. One phenotype was based on cluster analysis and included patients that were “uninflamed” or “reactive” lung injury. The second phenotype was based on latent class analysis and included patients who were either hypo-inflammatory or hyper-inflammatory. The third phenotype was based on etiology and included patients with either direct injury to the lung or indirect injury to the lung. There were no significant differences in these biomarkers or the microbiome in the subphenotypes in these three classifications. These results suggest that the phenotype classifications used in this study do not adequately reflect alveolar inflammatory responses. This greatly complicates efforts to use systemic biomarkers to identify phenotypes. In effect, most clinicians depend on clinical classifications to initiate treatment of patients with ARDS.

### 2.4. Clinical Classifications

The most obvious clinical calcifications include the primary disease process causing or associated with ARDS, which is usually classified as either pulmonary or extrapulmonary, the degree of hypoxemia based on a PaO_2_/FiO_2_ ratios, and the extent of lung involvement based on radiographic images, both plain chest radiographs and computed tomography. These classifications have the potential to lead to personalized care and often form the basis for trial design discussed below.

Chest radiographs are readily available and provide important information in critically ill patients but, of course, cannot identify the exact pathologic change(s) in individual patients. Chiumello et al. reviewed lung imaging techniques in patients with ARDS. Computed tomography provides a better description of the distribution of infiltrates and the density of infiltrates [24,25]. However, this technique requires the transfer of the patient to the radiology suite. Bedside ultrasonography is readily available and may have better accuracy than bedside chest X-rays in certain situations, such as pleural effusions. Other techniques that have been studied in ARDS patients include positron emission tomography, electrical impedance tomography, and magnetic resonance imaging. Chest radiographs, computed tomography, lung ultrasound, and electrical impedance tomography can be used to evaluate lung recruitment during PEEP trials. Patients with diffuse lung involvement on CT have higher mortality rates than patients with lobar or patchy involvement [24]. An increase in involvement during the early phase of ARDS is also associated with increased mortality. In addition, information collected on one day may not be accurate the following days because of the rapid progression of the inflammatory process and fluid accumulation.

Advances in technology have provided additional important information about the lung involvement and ARDS. Electrical impedance tomography uses a device with 16–32 electrodes to inject small amounts of electrical current around the chest [26]. The conduction of these currents is quantified to measure the level of tissue resistance. An air-filled lung has electrical resistance and increased impedance. A lung with a significant amount of extracellular water serves as a better conductor and has reduced impedance. These signals can be reconstructed into a digital matrix to generate a cross-sectional map of lung impedance through the respiratory cycle. Consequently, this device provides real-time information about lung ventilation. This information has been used to identify areas of lung overdistention and lung collapse. It can also be used to determine lung recruitment during PEEP trials and during prone positioning [27]. However, there is limited information demonstrating that the use of electrical impedance tomography managed ventilator adjustments changes outcomes.

**Figure 2:**
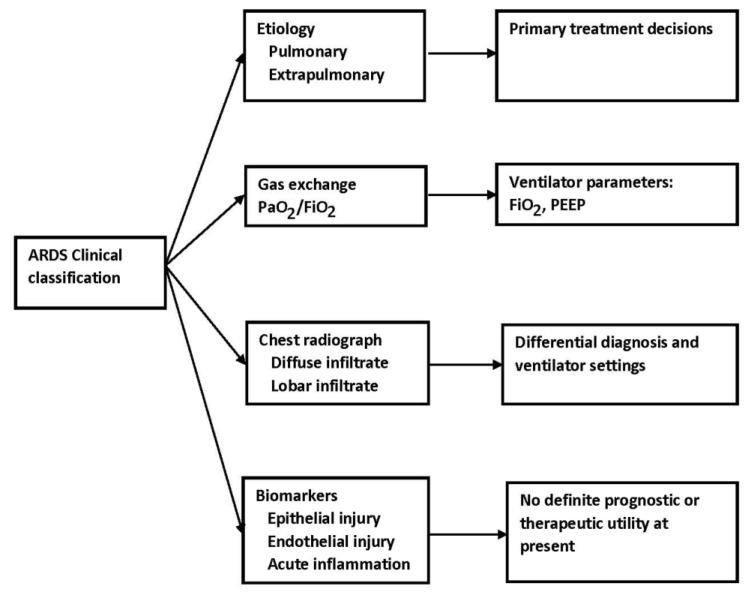
ARDS and clinical classification.

In summary, bedside techniques available to evaluate the patient’s lung include chest radiographs, ultrasonography, and electrical impedance tomography. Ultrasonography can be used to characterize the patient’s lung disease and to evaluate the effects of changes in PEEP levels on lung recruitment. It also provides information about the presence of pneumothorax and/or pleural effusions. These techniques offered the opportunity for personalized care or the adjustment of care based on individual characteristics (Figure 2).

### 2.5. Clinical Trial Outcomes

The outcomes in large well-designed treatment trials illustrate the difficulty in studying these patients. The first trial to report a mortality benefit in ARDS was the Acute Respiratory Distress Syndrome Network (ARDSNet) trial, which reported an 8.8% decrease in mortality (31% vs. 39.8%) in patients treated with low tidal volume ventilation (6 mL/kg vs. 12 mL/kg of ideal body weight) [28]. The Fluids and Catheters Treatment Trial (FACTT) demonstrated an increase in ventilator-free days but no change in mortality in patients treated with a conservative fluid management protocol, and the Proning Severe ARDS Patients (PROSEVA) trial reported decreased mortality with prone positioning in ARDS patients with a PaO_2_/FiO_2_ less than 150 mmHg [29,30]. By selectively enrolling patients with worse oxygenation (PaO_2_/FiO_2_ < 150 mmHg), the PROSEVA trial used a prognostic enrichment protocol, which selected patients with a higher likelihood of experiencing the primary study outcome. This increases the likelihood of detecting a difference in the outcome of interest with smaller sample sizes for a given effect size and provides a logical approach to develop inclusion criteria for clinical trials.

In contrast to the better outcomes based on optimal supportive care for ARDS patients, clinical trials of drug treatment have often had negative outcomes. The Dexamethasone Treatment for the Acute Respiratory Distress Syndrome: A Multicentre, Randomised Controlled Trial demonstrated that corticosteroid administration decreased mortality in patients who were treated early in their disease course; however, the Late Steroid Rescue study reported more adverse outcomes when corticosteroids were administered after 14 days of unresolving ARDS [31,32]. These two trials demonstrate that time-sensitive therapeutic windows likely influence the outcomes of drug trials and indicate the need for additional research to precisely define the stages of ARDS to optimize treatment.

The heterogeneity of ARDS pathophysiology and outcomes is also affected by differences in the treatment of these patients in ICUs. The 2000 ARDSNet trial reported decreased mortality with low tidal volume ventilation, whereas The Large Observational Study to Understand the Global Impact of Severe Acute Respiratory Failure study, published in 2016, reported that more than 33% of patients with ARDS were managed with a tidal volume of >8 mL/kg of ideal body weight [33]. There are several possible factors contributing to this undesirable variability in care, and these include delayed recognition of ARDS and overestimation of the necessary tidal volume in patients. In addition to the incomplete use of proven therapies, there are likely to be other variations in other treatment approaches in ARDS that have not yet been identified, and differences in available critical care resources could contribute to heterogeneity in care in ARDS patients. The management of large multi-center trials will require strict inclusion criteria, practical treatment protocols, and frequent communication among the investigators throughout the trial.

### 2.6. Trial Design

During the COVID-19 pandemic, critical care investigators responded to the need for new treatment by establishing large platform trials, such as I-SPY COVID (a multi-center phase 2 adaptive platform trial designed to rapidly screen potential therapeutics for severe COVID-19), the Accelerating COVID-19 Therapeutic Interventions and Vaccines (ACTIV) suite of trials, and Randomised Evaluation of COVID-19 Therapy (RECOVERY) [34,35]. These studies identified the beneficial effect of dexamethasone, baricitinib, and tocilizumab in COVID-19 ARDS and the lack of benefit with hydroxychloroquine and ivermectin. These trials had a different platform design, in which multiple potential therapies were tested simultaneously against a single control group, which can significantly increase trial efficiency compared to traditional randomized controlled trials that usually tests one therapy at a time. Of course, these studies require large patient numbers and complex study designs and cannot depend on precision medicine, at least at present. Adaptive trial design has the potential to improve outcomes in complex clinical disorders [36].

### 2.7. Summary

In summary, investigators have identified several subgroups of ARDS, based on the heterogeneous aspects of this disease process, including host factors, etiology and timing of injury, radiographic injury patterns, and disease severity. Certain phenotypes have different responses to drug treatment when evaluated retrospectively, which revealed the important finding that the uniform use of a drug in ARDS, without concurrent endophenotyping, may limit the ability of investigators both to detect benefits in certain phenotypes and to detect harm in others. More research is needed to understand the effect of anatomic and physiologic heterogeneity of ARDS due to both patient-specific factors and disease-specific factors, since these differences could have important effects on lung mechanics and ventilation strategies and could form the basis for precision medicine.

## 3. ARDS in Patients with COVID-19 Infection

The pandemic associated with SARS-CoV-2 infections has created crises in healthcare, the global economy, and education at all levels. However, this pandemic has created opportunities to study healthcare delivery and the management of critically ill patients with ARDS secondary to COVID-19. In contrast to patients with usual or typical ARDS, the cause of COVID-19-associated ARDS is quite clear [37,38]. This viral pathogen infects the respiratory tract and causes clinical presentations, which range from asymptomatic upper respiratory infections to acute respiratory failure requiring mechanical ventilation. The latter patients have bilateral infiltrates, severe hypoxemia, and variable changes in lung compliance [38]. Early studies describe patients with significant hypoxemia but minimal dyspnea (Chiumello) [39]. In addition, these patients often had higher levels of lung compliance than patients with the usual or typical ARDS. However, as more information accumulated, it became apparent that patients with ARDS associated with COVID-19 had a spectrum of pathophysiologic changes and that many patients had lung compliance levels similar to patients with typical ARDS [40]. The pathologic changes in patients with COVID-19 ARDS included diffuse inflammation, injury to epithelial and endothelial structures, diffuse alveolar damage, hyaline membrane formation, fibrosis, and diffuse microthrombosis [41]. Vascular pathology in these patients was much more pronounced than the vascular pathology in patients with typical ARDS [42]. This raised concerns about the use of anticoagulation in these patients to limit lung injury [43]. In addition, these patients often developed dysfunctional host defense responses, referred to as cytokine storms, and had multiorgan injury. Clinicians adjusted the mechanical ventilator parameters (i.e., tidal volume and PEEP levels) to fit the underlying pathophysiology based on static compliance measurements and gas exchange measurements (PaO_2_/FiO_2_). Some patients did well with higher tidal volumes and lower PEEP levels, but some patients required lower tidal volumes and higher PEEP levels. Many patients were treated with prone positioning and paralytic drugs [42]. Finally, patients with COVID-19 ARDS requiring mechanical ventilation had better outcomes with corticosteroid treatment than patients with typical ARDS [37].

## 4. Statistical Methods to Identify Phenotypes

The analysis of large existing databases of patients requiring mechanical ventilation using statistical methods can potentially identify phenotypic subtypes associated with either different outcomes or different management strategies. Ideally, these classification criteria should be available at the time the patient is admitted to the intensive care unit. Chen et al. retrospectively studied two large ICU databases, the eICU Collaborative Research Database (eICU) and the Medical Information Mart for Intensive Care (MIMIC-IV), to determine clinical phenotypes of ventilated ICU patients on the first day of ventilation [44]. For the derivation cohort, the K-means clustering model was used to identify clinical phenotypes using the eICU dataset. For the validation cohort, the clustering analysis results were then tested and validated using the MIMIC-IV dataset. The characteristics and clinical outcomes of different clinical phenotypes were then compared. A total of 15,256 mechanically ventilated patients from the eICU database and 10,813 from the MIMIC-IV database were included in this study.

Four clinical phenotypes were identified. The phenotype A cohort had 2175 patients who had a mortality rate of 23.4% and a successful extubation rate of 77.9%. These patients had higher median PaCO_2_ levels (53.3 mmHg; Q1, Q3; 47.6, 65.7). The phenotype B cohort had 2604 patients with a mortality rate of 34.0% and a successful extubation rate of 64.7%. These patients had higher median respiratory rates (22.9; Q1, Q3; 20.3, 26.1), median heart rates (99.4; Q1, Q3; 86.7, 112.1), and median WBC counts (14.3; Q1, Q3; 10.2–19.5). The phenotype C cohort included 2567 patients with a mortality rate of 40.1% and a successful extubation rate of 67.7%; these patients had higher median creatinine levels (2.4; Q1, Q3; 1.6, 3.8). The phenotype D cohort had 3467 patients with a mortality rate of 19.4% and a successful extubation rate of 82.1%. These patients have the lowest median Glasgow Coma Scale Score (8.1; Q1, Q3; 6.3, 9.8). There was no relationship between these clinical phenotypes and APACHE IV scores, which suggested that they do not simply reflect the disease severity. Patients in the phenotype B group required higher PEEP levels (median 8.2 cm H_2_O; Q1, Q3; 5.7, 10.0), and these patients had statistically significant lower extubation success rates, longer ventilation times, and longer ICU stays, but had no significant differences in hospital mortality. Determining the clinical phenotypes of mechanically ventilated patients might help intensivists predict 28-day mortality, extubation success rates, the duration of mechanical ventilation, and the length of ICU stays. This could help intensivists make clinical decisions to personalize treatment strategies and improve discussions with patients and their families.

This study has several limitations. First, only the data on the first day of intubation were analyzed in this study. The complex and dynamic disease trajectory in the majority of critically ill patients may result in changes in the clinical phenotype after the first day, and consequently, the clinical phenotype on the first day of intubation might not represent the “correct” phenotype over the course of illness for some patients. In addition, classification of patients based on abnormal clinical and laboratory data on the day of admission to the hospital will require a clinical tool (score) to summarize the important variables, since no single parameter can identify patients with any level of certainty. Second, weaning guidelines were updated in 2016, and since the data from the MIMIC database were from 2008 to 2019 and the data from the eICU were from 2014 to 2015, the weaning process might vary among patients, institutions, and clinicians, resulting in different extubation success rates, ventilator times, and ICU stays. Future studies should include and analyze data on the first day of intubation and possibly day 3 after intubation to determine the clinical trajectory and possibly a more accurate phenotype classification. A dataset collected after 2016 should be studied to determine if more recent guidelines might improve extubation success rates, ventilation duration, and ICU stays. In addition, the analysis of different approaches to weaning might identify more successful strategies or identify strategies specific to the underlying cause of respiratory failure.

Machine learning has been used to analyze large ARDS databases to determine features associated with responses to particular treatment. Sinha et al. compared machine learning clustering algorithms to detect the heterogeneity of treatment effects in ARDS, using results from three randomized controlled trials [45]. Five unsupervised clustering algorithms and four supervised algorithms were studied. The information used in these classifications included clinical data and protein biomarkers. The main effort was to determine whether or not they could identify the heterogeneity of treatment effects, which would support treatment decisions. None of the algorithms identified clusters with significant heterogeneity of treatment effects in all three trials. They also determined that the results from the algorithms depended on the starting point from which the algorithm was generated, the “initialisation of seed”, and biomarkers were essential to identify the heterogeneity of the treatment effect. These authors concluded that investigations using machine learning approaches to identify patients who might benefit from particular treatment require “cautious interpretation”.

## 5. Treatment Goals

**Figure 3:**
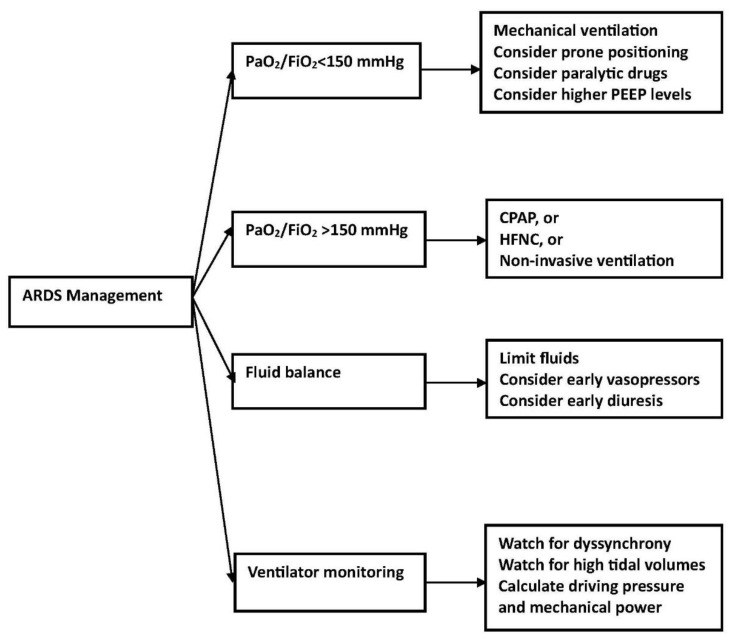
ARDS and initial management.

Using precision medicine, the investigator can identify the basic physiologic process in the patient causing acute lung injury and will know the medications available, if any, to stop the injury and reverse lung damage. At present, the clinician will not have the information necessary to practice precision medicine but will make use of clinical trial results that identified certain subphenotypes that had better outcomes with a particular management strategy. This would represent personalized medicine, and at present, this approach largely involves adjustments in mechanical ventilation parameters. This will likely require radiographic and physiologic characterization, which leads to decisions regarding lung recruitment, PEEP level, patient positioning, the use of particular sedatives and narcotics, the use of paralytic drugs, and the use of fluid and diuretics. This characterization will need a more precise estimate of extravascular lung water and management strategies, which involves fluid administration, vasopressor use, and diuretic use (Figure 3).

## 6. Initial Treatment Decisions

### 6.1. Non-Invasive Ventilation (CPAP) in COVID-19 Infection

After the patient is admitted to the ICU, clinicians must make decisions about the type and level of respiratory support and treatment of the primary disorder, if known. Many patients with ARDS require intubation and mechanical ventilation, and, at present, this approach provides the best respiratory support available to critically ill patients. However, prolonged endotracheal intubation exposes patients to several potentially important complications, including central airway injury, nosocomial infection, and adverse drug effects used to limit pain and anxiety, which can cause both acute and chronic side effects. Can non-invasive ventilation provide adequate respiratory support for some patients with ARDS? Do hospitals need to develop specialized units to provide this level of care?

Coronavirus disease 2019 affected several million people worldwide and challenged clinicians and critical care units to provide more and better patient care. Non-invasive respiratory systems (NIRS) were often used as an intermediate level of support in specialized respiratory care units in an effort to avoid invasive mechanical ventilation. Accordino et al. analyzed the characteristics and outcomes in patients admitted to the intermediate care unit with acute respiratory failure due to COVID-19 and managed with continuous positive airway pressure (CPAP) through the four waves of the pandemic [46]. Acute respiratory failure was defined as PaO_2_/FiO_2_ < 300 mmHg or PaO_2_ < 60 mmHg. Patients who tested negative for SARS-CoV-2 by PCR, who had ARF due to a cause other than COVID-19 pneumonia, and who required mechanical ventilation at any time during the hospital course of COVID-19 pneumonia were excluded from the study. The study included 300 patients admitted to the center during the four COVID-19 waves from March 2020 to April 2022 in Italy.

The study provides information about the characteristics of COVID-19 patients who were managed by CPAP throughout their hospital stay. These patients had a median age of 73, a median PEEP level on CPAP of 7.5 cm H_2_O (Q1, Q3; 5, 15), a median FiO_2_ on CPAP of 0.50 Q1, Q3; 0.3, 0.95), a median admission PaO_2_ of 67 mmHg, PaCO_2_ of 33 mmHg, and PaO_2_/FiO_2_ ratio of 134 mmHg. These patients were managed on CPAP for a median of 8 days (Q1, Q3; 1, 96), 10.7% required ICU transfer, and the overall mortality was 36.3%. These results suggest that the use of CPAP in a highly specialized respiratory care unit can provide adequate support for a critically ill subset of patients with COVID-19 pneumonia and presumably patients with ARDS unrelated to COVID-19 infection.

### 6.2. Studies with Non-Invasive Ventilation in Non-COVID-19 ARDS

Should all patients with acute respiratory failure with a likely diagnosis of ARDS be initially managed with non-invasive ventilation? This approach prevents the complications associated with intubation and the prolonged use of endotracheal tubes. Bellani et al. analyzed the use of non-invasive ventilations in patients with acute respiratory distress syndrome in the Lung Safe study [47]. This study included 2813 patients with ARDS; 436 patients were managed with non-invasive ventilation on days 1 and 2. This approach failed in 22.2% of patients with mild ARDS based on the PaO_2_/FiO_2_ ratio, in 42.3% of patients with moderate ARDS, and in 47.1% of patients with severe ARDS. Hospital mortality in patients with NIV success was 16.1%, and hospital mortality was 45.4% in patients with NIV failure. ICU mortality was higher in patients managed with NIV than invasively ventilated patients with a PaO_2_/FiO_2_ ratio less than 150 mmHg.

Carteaux et al. analyzed the outcome of patients with new onset acute hypoxemic respiratory failure not explained by cardiac failure who were managed with non-invasive ventilation [48]. This study included 62 patients; 32 patients failed non-invasive ventilation. The median expired tidal volume in all non-invasive ventilation sessions was 9.8 mL per kilogram predicted body weight, and the tidal volume was significantly higher in patients who failed non-invasive ventilation. This effect was most apparent in patients with a PaO_2_/FiO_2_ ratio of less than 200 mmHg. In multivariate analysis, factors associated with failure included the Simplified Acute Physiology Score 2 and the mean expired tidal volume. Possible explanations include more severe respiratory disease and increased respiratory drive in patients with higher tidal volumes. In addition, higher tidal volumes may produce volutrauma related to increased transpulmonary pressures.

Aswanetmanee et al. recently reported a systematic review and meta-analysis of randomized controlled trials that compared non-invasive ventilation with conventional oxygen therapy, including oxygen by mask and high-flow nasal cannulas [49]. The risk for tracheal intubation was lower in patients managed with non-invasive ventilation. However, there was no difference in the ICU or hospital-related mortality. Non-invasive ventilation did not result in a significant reduction in intubation when compared to high-flow nasal cannula support alone in two studies included in this review. The use of non-invasive ventilation does have a potential risk associated with increased tidal volumes and increased transpulmonary pressures causing lung injury. Rochwerg et al. summarized the guidelines developed by the European Respiratory Society and the American Thoracic Society on the use of non-invasive ventilation for various causes of acute respiratory failure [50]. These experts did not make a recommendation on the use of non-invasive ventilation in new onset acute respiratory failure given the uncertainty of the evidence. They did suggest that a trial of non-invasive ventilation might be used in patients with hypoxemic respiratory failure, pneumonia, or early ARDS, if they were managed by an experienced clinical team and there were no contraindications, such as altered mental status or multiorgan failure.

In summary, non-invasive ventilation provides adequate support for many patients with acute hypoxemic respiratory failure. These patients should be managed in an experienced respiratory care unit with frequent reassessment. Delayed intubation has been associated with worse outcomes. Patients with a PaO_2_/FiO_2_ ratio less than 150 mmHg are candidates for initial intubation and mechanical ventilation. Patients with a PaO_2_/FiO_2_ ratio greater than 150 mmHg are potentially candidates for non-invasive ventilation or high-flow nasal cannula oxygen supplementation. These patients need to be monitored closely for changes in mental status, respiratory muscle fatigue, and high tidal volumes that could cause lung injury.

### 6.3. Fluid Management in COVID-19 Infection

Lung injury in ARDS involves both capillary and epithelial structures. Capillary injury increases permeability, which allows the transfer of fluid out of the vessels into the interstitium and alveoli at lower hydrostatic pressures. Fluid management in these patients can be perplexing and requires considerations about intravascular volume and tissue perfusion and extravascular fluid accumulation. Several studies have tried to compare the differences in outcome between restrictive and liberal fluid administration. Is it possible that cautious diuresis at the onset of illness has benefits and different outcomes compared to strategies that try to limit fluid balance over multiple days? A small study developed during the management of patients with COVID-19 respiratory infection provides very interesting results on the potential benefits of negative fluid balances [51].

Cytokine storm is an important pathophysiologic process in COVID-19 and contributes to the development of ARDS and the long-term sequelae after this infection [51]. However, there are several other proposed mechanisms responsible for lung injury and the development of ARDS in COVID-19 patients; these include increased inflammatory responses, oxidative stress, and renin–angiotensin–aldosterone system (RAAS) dysregulation. SARS-CoV-2 downregulates ACE2 expression, resulting in aldosterone upregulation and RAAS activity dysregulation. This increases sodium and water reabsorption, subsequently leading to volume overload and possibly hypoxemia. Francisco Santos et al. suggest that the pathophysiology of severe acute pulmonary edema in COVID-19 is a multiple-step process [51]. Initial pneumonitis causes increased alveolar–capillary membrane permeability, resulting in low hydrostatic pressure pulmonary edema. This leads to high pressure pulmonary edema, caused by increased hydrostatic pressure secondary to volume overload as a result of RAAS dysregulation. If the pulmonary edema does not resolve, and the patient has prolonged respiratory distress, more inflammation, and typical ARDS can occur.

Francisco Santos et al. conducted a single-center retrospective observational study in 20 critically ill COVID-19 patients with PaO_2_/FiO_2_ < 200 mmHg and tomographic evidence of pulmonary edema (dilated superior vena cava [SVC], large pulmonary arteries, diffuse interstitial infiltrates with Kerley lines, and dilated right ventricle or dilated cardiac axis) who received continuous intravenous (IV) furosemide infusion [51]. This negative balance protocol included oral fluid restriction and 20 mg of furosemide IV bolus, followed by continuous IV furosemide infusion, starting at 60 mg/day. The goal was to achieve a negative fluid balance between 600 and 1400 mL/day with a final target of 8–10% of body weight in 8 days. The relationship between the variation in the PaO_2_/FiO_2_ ratio and the variables on admission to this protocol and on day 7 were analyzed using a linear regression model.

Two patients in this cohort died, one from sepsis and one from acute myocardial infarction. The average dose of IV infused furosemide was 3.5 ± 2 mg/h on day 1, 4.2 ± 2 mg/h on day 2, 4.5 ± 3 mg/h on day 3, 4.5 ± 3 mg/h on day 4, 4.8 ± 3 mg/h on day 5, 4.8 ± 4 mg/h on day 6, and 5.5 ± 4 mg/h on day 7. The mean total accumulated negative fluid balance was −7637 mL (±2616). None of the 20 cases developed electrolytes or serum creatinine alterations or hypotension during negative fluid balance management. TH indirect parameters analyzed during this study included the diameter of the SVC, the length of cardiac axis, anemia, heart rate, CT score, PaO_2_/FiO_2_ ratio, and fluid balances that could reflect volume overload secondary to RAAS dysregulation in COVID-19 infections.

Oxygenation improved in all cases with a significant increase in the PaO_2_/FiO_2_ ratio on day 4 and day 8 (mean 246 ± 111 mmHg and 316 ± 90 mmHg, respectively) compared to the values obtained upon admission for the PaO_2_/FiO_2_ ratio (mean 118 ± 47 mmHg). Computed tomography scores improved significantly on day 4 and day 8 (mean 10.6 ± 1.6 and 7.7 ± 1.4, respectively, compared to the score on admission in the study (mean 17.2 ± 1.3). The hematocrit level at discharge compared to upon admission increased significantly from 37.5 ± 4.9% to 40.7 ± 5%. The diameter of the superior vena cava at discharge compared to admission decreased significantly from 17.9 ± 3.8 to 13.9 ± 3 mm.

There were no clinical signs of systemic inflammatory response syndrome or increases in leukocyte counts and C-reactive protein levels in this study; this suggests that cytokine storm might not have an important role in the early stages of COVID-19 over the clinical course at times in which volume overload may have important physiologic effects secondary to leaky capillaries. This study has several limitations. It is a retrospective, observational study, so there was no control group, and only 20 patients were included. Francisco Santos et al. published a second paper on patients with COVID-19 infection who were managed with a similar strategy in their ICU and compared this group of patients with patients who did not undergo the negative fluid balance protocol [52]. The identification of the two groups was based on the date on which the negative balance protocol was introduced; both arms included 58 patients. Compared to the control patients, the patients with a negative fluid balance had a significantly lower mortality, fewer hospital days, fewer ICU days, and fewer days of mechanical ventilation. They had significant improvements in the PaO_2_/FiO_2_ ratio and in their composite CT score, which reflects the fluid status in the thorax. The early phase of clinical disorders associated with ARDS is characterized by increased capillary permeability, which results in pulmonary edema even at low hydrostatic pressures and this early interstitial and alveolar edema causes more inflammation and lung injury.

### 6.4. Fluid Management in Non-COVID-19 ARDS

The results from these two COVID-19 studies should be compared to a very large study published in 2006, which compared restricted versus liberal fluid management strategies in a multi-center study. Wiedemann et al. [29] compared two fluid management strategies in patients with acute lung injury using explicit and detailed protocols for the first seven days of management in these patients. The most frequent cause of lung injury in these patients was pneumonia. The mean cumulative fluid balance during the first day for seven days was −136 ± 491 mL in a conservative strategy group and +6992 ± 502 mL in the liberal strategy group. The fluid balances were positive in both groups on day 1 of this study. The patients in the conservative strategy group had improved oxygenation, lung injury scores, and more ventilator-free days but no reduction in mortality at 60 days. They did not have more frequent shock or require more dialysis. There are important differences between the studies done with the COVID-19 patients and this particular study, especially in fluid balance on day 1 of ICU care. Consequently, early management of fluid administration starting on day 1 of ICU admission could have important effects in patients with typical ARDS. This possibility obviously needs more study and would require frequent attention to blood pressure and organ perfusion and possibly the early use of vasopressors.

### 6.5. The Potential Hazards Associated with Positive Fluid Balances

These studies in one ICU with COVID-19 patients would suggest that early events during the development of interstitial and alveolar edema may trigger or amplify inflammatory processes associated with the primary diagnosis. Imai and coworkers have analyzed the renin–angiotensin system and acute respiratory distress syndrome [53]. They note that multiple acute events, including pneumonia and sepsis, can activate this system and generate angiotensin II. The binding of this molecule to the angiotensin I receptor can cause severe inflammation and increase vascular permeability. This pathway could represent an early event in acute respiratory distress syndrome. Hippensteel et al. studied the degradation of the endothelial glycocalyx in patients with sepsis by measuring plasma heparan sulfate levels [54]. These levels were increased in patients with septic shock by 6 h after presentation to the emergency department and continued to increase over the next 24 h. The level of this marker was associated with plasma biomarkers for endothelial injury, coagulation, and inflammation. The level at 6 h was independently associated with the cumulative intravenous fluid balance over these initial 6 h in a multivariate model. These authors suggested that fluid resuscitation could cause iatrogenic glycocalyx degradation secondary to volume overload. Another possible mechanism involved endothelial shear stress caused by fluid boluses. Increasing endothelial injury would increase permeability and increase extravascular fluid in the lungs.

### 6.6. Measurement of Extravascular Fluid

To study this possibility requires early detection of extravascular lung water. Tagami et al. reviewed the measurement of extravascular lung water in acute respiratory distress syndrome and discussed the measurement extravascular lung water and pulmonary vascular permeability using a transpulmonary thermodilution technique [55]. These two calculations allow clinicians to determine whether patients have cardiogenic pulmonary edema or non-cardiogenic pulmonary edema. Additional measurements with this system include cardiac output, global end-diastolic volume, and global ejection fraction, which provide important information about the cardiovascular status. Serial measurements should help determine disease progression and responses to treatment. Consequently, the technology is available to determine whether patients have developed interstitial and alveolar edema early in their hospital course.

Kushimoto et al. measured extravascular lung water and pulmonary vascular permeability in 266 patients with a PaO_2_/FiO_2_ ratio less than 300 mmHg and bilateral infiltrates on chest radiographs [56,57]. Three expert clinicians retrospectively reviewed clinical information to determine if the patients had ARDS, cardiogenic pulmonary edema, or atelectasis with pleural effusion. Extravascular lung water and pulmonary vascular permeability were higher in patients with ARDS than in patients with cardiogenic pulmonary edema and in patients with atelectasis and pleural effusion. Extravascular lung water increased with increasing pulmonary vascular permeability, and increased permeability indices can definitively diagnose ARDS. Komiya et al. reviewed the literature to determine the utility of biomarkers in trying to differentiate acute respiratory distress syndrome from cardiogenic pulmonary edema [58]. The most frequently used biomarker was the brain natriuretic peptide, and this biomarker provided relatively good discrimination between these two conditions when measured early in the clinical presentation. However, the cutoff values for a positive test ranged from 100 to 500 pg/mL in four studies, and this suggests that this biomarker is unlikely to adequately differentiate patients with acute respiratory failure from patients with acute cardiogenic pulmonary edema. Consequently, direct hemodynamic measurements may be necessary to correctly classify patients.

## 7. Physiological Monitoring during Mechanical Ventilation

Most mechanical ventilators provide minute to minute information about the patient’s respiratory status. Simple measurements include the respiratory rate, peak pressure, plateau pressure, and driving pressure. Clinicians usually review this information during discrete time intervals during patient assessment, and it is recorded by other healthcare workers in the patient’s medical records. Information about early trends in these parameters, such as a steady reduction in respiratory rate, might provide predictions about the patient’s outcomes and trigger ventilator adjustments. In addition, patients requiring mechanical ventilation often develop patient–ventilator asynchrony in which the patient’s respiratory system demand is not matched by the ventilator performance [59]. Common examples of asynchrony include ineffective triggering in which the patient tries to trigger the ventilator, but no volume is delivered, and double triggering in which a second breath follows the proceeding breath without adequate time for exhalation. These events can cause anxiety, dyspnea, increased respiratory muscle work, muscle injury, decreased sleep quality, and increased requirement for sedation and/or paralytic medications [60]. Ventilator asynchrony can also lead to ventilator-induced lung injury, prolonged mechanical ventilation, and difficulty weaning. Physicians may not be aware that patients are having ventilator asynchrony and usually do not know the frequency of the asynchrony, the type of asynchrony, and the potential adverse effects associated with any ongoing asynchrony. Current ventilators provide important information about the respiratory system, including respiratory frequency and flow–time parameters, pressure–time parameters and volume–time parameters. More advanced analysis of these parameters might identify asynchrony and potentially function like respiratory telemetry.

Su and colleagues developed a remote mechanical ventilation visualization network system and an automatic recognition algorithm to analyze respiratory patterns and identify ventilator asynchrony [61]. Their system included data collection, data processing, and data reports. The technology required to undertake this analysis is quite complicated and labeled data are needed to train the model to identify episodes of ventilator asynchrony. Eventually, the analysis creates reports and could be connected to an alarm system to alert clinicians to ongoing problems during patient care. They reported information from four patients. The results included 4496 breaths with 716 double triggering events and 910 ineffective triggering events. The algorithm had a sensitivity of 67% detecting double triggering events and 80% predicting ineffective trigger events; the algorithm detected ineffective triggering at a higher rate than double triggering. The computer then created a report including numerical information and graphic displays.

The information collection by this system has two obvious applications. This system can provide information about the frequency of ventilator asynchrony, the type of ventilator asynchrony, and the potential consequences/associations, including ventilation time and adverse outcomes during mechanical ventilation. Answering these questions will require large-scale studies with heterogeneous groups of patients, but the technology and automatic analysis of data make this relatively easy to do in most ICUs. If studies can determine the frequency of asynchronous events that are associated with adverse outcomes, then a warning system can be developed to alert clinicians that the patient has an unsafe number of asynchronous events. A simpler application of this system could involve measurement of respiratory rates and trends and analysis of respiratory rhythms.

## 8. Conclusions

The literature summarized in this review has several important implications for the initial management of patients with ARDS. Developing a phenotypic classification of patients with acute respiratory failure using statistical classification methods has potential to more effectively identify factors relevant to outcomes but probably will not provide a classification of patients during their initial management because it is unlikely that one or two clinical or laboratory parameters that are readily available at the bedside will provide a distinct separation of patients into subgroups. Therefore, precision medicine is not yet available for these patients. Personalized care is available but this largely represents making management decisions based on the individual characteristics of the patient. Important initial decisions include whether or not to use non-invasive ventilation in patients with ARDS and to establish negative fluid balances early in the treatment course. Continuous positive airway pressure and non-invasive ventilation can provide adequate respiratory support for some patients with acute respiratory failure; this approach might require the development of specialized respiratory care units. The creation of a negative fluid balance early in the course of a patient with acute respiratory failure that will likely evolve into ARDS may change the pathophysiology and the prognosis. This approach to fluid management would require early identification of pulmonary edema in patients and then fluid restriction and possible diuresis. Finally, sophisticated algorithms to identify episodes of dyssynchrony have the potential to alert clinicians to difficulties with patient–ventilator interactions. However, these efforts would require the manufacturers to create the software necessary for this application, since it is unlikely that individual institutions or ICUs could do this, especially since these algorithms require training.

## Data Availability

Not applicable.
